# Hyperventilation during rest and exercise in orthostatic intolerance and Spiky-Leaky Syndrome

**DOI:** 10.3389/fneur.2025.1512671

**Published:** 2025-04-17

**Authors:** Amir Hashemizad, Jerriel Dela Cruz, Aditya Narayan, Andrew J. Maxwell

**Affiliations:** Heart of the Valley Pediatric Cardiology, Pleasanton, CA, United States

**Keywords:** dysautonomia, POTS, mast cell activation syndrome, Ehlers-Danlos syndrome, hypermobility spectrum disorder, Spiky-Leaky Syndrome, end-tidal CO_2_, exercise stress testing

## Abstract

**Background:**

Orthostatic intolerance, with or without postural orthostatic tachycardia syndrome (POTS), is collectively referred to as orthostatic intolerance dysautonomia syndromes (OIDS). This condition often presents with daytime hyperventilation, which is considered to be secondary to sympathetic hyperactivity. This hyperventilation appears to be a key characteristic in a newly described subset of patients with OIDS who also exhibit craniocervical instability, mast cell activation syndrome (MCAS), hypermobility spectrum disorder (HSD), and the phenomenon of alternating intracranial hypertension with hypotension due to cerebrospinal fluid (CSF) leaks, collectively termed Spiky-Leaky Syndrome (SLS).

**Methods:**

We performed a retrospective review of clinical metabolic exercise data in young patients with SLS, comparing them to matched patients with OIDS and healthy controls (CTL). We assessed metabolic parameters at rest, at the anaerobic threshold (AT), and at maximal oxygen consumption (VO_2_max). The parameters included end-tidal CO_2_ (ETCO_2_), end-tidal O_2_ (ETO_2_), peak oxygen pulse, total work performed, and peak oxygen uptake efficiency slope (OUESp).

**Results:**

Of 323 reviewed exercise stress tests, 44 were conducted on patients with SLS, 210 on those with OIDS, and 53 on healthy controls. VO_2_max, AT, peak oxygen pulse, total work performed, and OUESp were all significantly reduced in patients with OIDS and were further reduced in those with SLS. ETCO_2_ levels were notably lower at rest, at the time of the anaerobic threshold, and at the time of maximal oxygen uptake in the OIDS group, and even more so in the SLS group. These lower levels of ETCO_2_ persisted throughout exercise. In contrast, ETO_2_ demonstrated a similarly strong but opposite trend.

**Conclusion:**

Compared to the control group, patients with OIDS—and especially those with SLS—exhibited reduced metabolic parameters, particularly a decrease in peak oxygen pulse and ETCO_2_ levels during both rest and exercise. These findings suggest a reduction in ventricular preload and chronic daytime hyperventilation. These exercise parameters may serve as markers for POTS physiology and sympathetic hyperactivity, both of which could play a role in the pathophysiology of SLS.

## Introduction

Patients with parasympathetic dysfunction often experience orthostatic intolerance (OI) as a key symptom. This includes individuals who have postural orthostatic tachycardia syndrome (POTS) and those who have chronic OI who experience a modest or minimal rise in orthostatic heart rate (HR)—usually without orthostatic hypotension but with other indicators of autonomic dysfunction. These indicators often include symptoms related to the cardiovascular system and presentations that overlap with myalgic encephalomyelitis/chronic fatigue syndrome (1). This population of patients that is broader than that defined by POTS has been described by Wheeler, Raj, and Boris (1, 22, 41). For the purpose of this report, we grouped these presentations under the term “orthostatic intolerance dysautonomia syndromes” (OIDS).

Patients with OIDS often develop chronic hyperventilation over the course of their illness, resulting in respiratory alkalosis. Stewart and Pianosi found that, at least in patients with POTS, this is mainly due to an increase in tidal volume with little change in the respiratory rate (2). They postulated that this may be linked to enhanced sympathetic activity, potentially due to altered carotid body responses to intermittent hypoxia, acidosis, and hypoperfusion, which could be exacerbated by significant fluctuations between hypercarbia and hypocarbia (3–5). Other researchers have postulated that reduced ventricular stroke volume and cerebral hypoperfusion more directly trigger hyperpnea (6). In patients with chronic OI (with or without POTS), daytime measurements of end-tidal carbon dioxide (ETCO_2_) often reveal values well below the standard set point of 40 mmHg (1). Several investigators have shown that chronic hypocarbia can reduce cerebral blood flow—particularly in the upright position—indicating a possible explanatory mechanism behind symptoms such as dizziness, syncope, brain fog, and migraine headaches (1, 4, 7–11).

Clinically, we have observed that hypocarbia, as measured through the surrogate measure of hypocapnia, can be assessed both at rest and during exercise by monitoring ETCO_2_ levels. Furthermore, measuring ETCO_2_ during exercise—particularly at key points such as the anabolic threshold (AT) and maximal oxygen uptake (VO_2_max)—can help reduce the influence of anxiety-related fluctuations at rest. Thus, combined resting and exercise-based ETCO_2_ data may offer a reliable measure of the presence and severity of hyperventilation, which could, in turn, serve as a marker of sympathetic hyperactivity.

Recently, we identified a new phenotype in the clinical setting characterized by having OIDS (in any of its presentations listed above) along with hypermobility spectrum disorder (HSD)—sometimes including hypermobile Ehlers-Danlos syndrome—mast cell activation syndrome (MCAS), evidence of craniocervical instability, upper airway resistance syndrome (UARS) at night, and chronic intermittent shifts between intracranial hypertension and hypotension. The latter can be associated with transcranial cerebrospinal fluid (CSF) leaks, which may manifest in some cases as dribbling from the nose or down the back of the throat or, in other cases, as a gush of fluid from the nose upon rising in the morning (12–14). We refer to this phenotype as “Spiky-Leaky Syndrome” (SLS), highlighting the nighttime spikes in CSF pressure and occasional CSF leakage through the ends of cranial nerve sheathes, most notably through the cribriform plate into the nasal mucosa when CSF pressure surpasses a modest pressure threshold. This phenotype has also been independently described more recently by two other groups (15, 16) and further discussed in a recent review (17).

We reviewed the most recent patients with OIDS in the corresponding author’s clinics to identify a cohort meeting the formal diagnostic criteria for POTS. This required an evaluation of 392 patients with OIDS, of whom 100 (25%) met the formal POTS criteria. Among the total OIDS population, 39 patients (10%) exhibited anatomical features associated with Spiky-Leaky Syndrome (SLS), including craniocervical instability, upper airway resistance syndrome, and jugular venous compression, but lacked evidence of cerebrospinal fluid (CSF) leakage. An additional 14 patients (4%) met the full diagnostic criteria for SLS.

Within the subset of 100 patients diagnosed with POTS, 78 had mast cell activation syndrome (MCAS), 33 had hypermobility spectrum disorder (HSD), and 33 exhibited anatomical features associated with SLS but without the evidence of CSF leakage. Of these, nine patients had fully developed SLS. Consequently, nine of 14 patients (65%) with SLS also met the formal POTS criteria. The relatively high prevalence of SLS among patients with POTS may be influenced by referral patterns unique to the clinics’ locations and specialty of the provider, potentially introducing referral bias (14, 42).

A key proposed mechanism underlying SLS involves exaggerated fluctuations in carbon dioxide levels, with daytime hypocarbia resulting from chronic hyperventilation—likely induced by sympathetic hyperactivity—and nighttime hypercarbia resulting from compromised airway patency, which is a common feature in individuals with HSD. As a result, individuals with SLS are expected to exhibit increased daytime hypocarbia, indicated by end-tidal carbon dioxide (ETCO_₂_) levels during exercise, compared to those with OIDS. This study investigated the degree of daytime hypocarbia in SLS, using ETCO_₂_ levels as a surrogate measure, and compared these findings with data from both OIDS patients and healthy controls.

## Methods

### Study participants

We reviewed clinical exercise testing data from patients aged 9 to 29 years who exhibited orthostatic intolerance (OI), as defined by Stewart (18) and Sandroni (9), along with at least one additional sign or symptom of autonomic nervous system dysfunction. These patients were evaluated by the corresponding author (AJM) at five cardiology centers between 2014 and 2024, with records selected from the patients referred to any of these clinics in the San Francisco Bay Area. The eligibility criteria included a history of orthostatic intolerance lasting at least 6 months, accompanied by at least one symptom such as syncope, palpitations, tachycardia, dizziness, fatigue, exercise intolerance, shortness of breath, or chest discomfort. Non-autonomic cardiopulmonary causes for these symptoms had to be ruled out through comprehensive clinical evaluation and ancillary testing. Patients were included if they met the criteria for chronic orthostatic intolerance, with or without fulfilling the formal diagnostic criteria for postural orthostatic tachycardia syndrome (POTS), as defined by the Pediatric Writing Group of the American Autonomic Society and the 2015 Heart Rhythm Society Expert Consensus Statement. In addition, patients meeting the diagnostic criteria for myalgic encephalomyelitis/chronic fatigue syndrome (ME/CFS), with or without POTS, based on the International Chronic Fatigue Syndrome Study Group definition, were included (19–21).

Patients who did not meet the POTS or ME/CFS criteria but reported chronic orthostatic intolerance with at least one additional symptom of autonomic dysfunction persisting for 6 months or longer were also included (22). The exclusion criteria included patients with OI symptoms lasting less than 6 months, cases where symptoms were attributable to medications or other identifiable illnesses known to cause OI, and instances where evaluation, identification, or resolution of the condition was brief and did not justify further assessment with validated autonomic function questionnaires such as COMPASS-31 (23). In addition, patients identified as having a post-COVID condition were excluded from the study. Those with incomplete exercise testing or an incomplete set of parameters were also excluded.

The patients were diagnosed with Spiky-Leaky Syndrome (SLS) based on the established phenotypic description (14). This diagnosis required the presence of OIDS alongside mast cell activation syndrome (MCAS), hypermobility spectrum disorder (HSD), and radiological and/or clinical evidence of craniocervical instability (CCI). Furthermore, the patients with SLS exhibited upper airway resistance syndrome (UARS), as confirmed by sleep studies incorporating continuous CO_2_ monitoring, specifically using end-tidal CO_2_ (ETCO_2_) and/or transcutaneous CO_2_ measurements throughout the study (14).

The control group consisted of healthy individuals who underwent exercise stress testing for reasons such as sports participation, employment clearance, or minor cardiac concerns. No individuals were excluded due to cultural or language barriers, and no participants received compensation for their involvement. A larger clinical database was compiled, from which a statistically balanced dataset was generated. The participants were matched based on age, weight, BMI, and sex, while the selection process remained blinded to ensure an unbiased final matched dataset.

### Exercise testing

Patients underwent metabolic exercise testing (Ultima CPX Metabolic Stress Testing System, MCG Diagnostics) with either a graded treadmill protocol (Modified Bruce Protocol, with step parameters adjusted for age) or a bicycle ergometer protocol (Continuous Ramp, with age-appropriate workload increments). Throughout the test, all patients were monitored via continuous pulse oximetry, electrocardiogram (ECG) recording, and intermittent blood pressure measurements. A headgear-mounted mask was used to measure the levels of end-tidal oxygen (ETO_2_) and end-tidal carbon dioxide (ETCO_2_). The exercise continued until the patients either voluntarily terminated the test or the operator determined that discontinuation was necessary for safety. Pulmonary function testing was performed before and after exercise.

The key metabolic parameters measured included ETCO_2_, ETO_2_, oxygen consumption at the anaerobic threshold (AT) and maximum effort (VO_2_max), peak oxygen pulse, total work performed, and peak oxygen uptake efficiency slope (OUESp). The anaerobic threshold (AT) was determined using the V-slope method developed by Wasserman (24–26). In the event that two inflection points occurred (i.e., AT_1_ and AT_2_), the more marked inflection point was selected. Peak oxygen pulse was derived by dividing VO_2_ by the heart rate (HR) at any given time, serving as a surrogate measure of stroke volume (27, 28). According to the Fick Principle, left ventricular (LV) stroke volume is calculated using the equation VO_2_ / (HR × AVO_2_ × Hbg × 1.36), where AVO_2_ represents the arteriovenous oxygen difference and Hbg denotes hemoglobin concentration (29). Given that stroke volume increases significantly during exercise while AVO_2_ undergoes relatively minor changes, oxygen pulse can reliably serve as a surrogate for stroke volume, measured in mL O_2_ per beat. The correlation between oxygen pulse and stroke volume has been reported with an R-value of approximately 0.73 (27). This parameter is typically reported at its peak value during exercise.

The peak oxygen uptake efficiency slope (OUESp) was calculated based on the linear relationship between oxygen consumption (VO_2_) and the logarithm of minute ventilation (VE), represented by the equation VO_2_ = OUIS × log(VE) + b, where b is a constant (30). This value was normalized to the patient body surface area (BSA) and reported at its peak (31). The OUESp has been validated as a reliable, effort-independent surrogate for VO_2_max, with a normal reference range of 1,000 to 2,200 mL O_2_/min/m^2^ BSA (31, 32).

### Statistical approach

Statistical analyses were conducted using IBM® SPSS Statistics (Version 29) and Python’s scientific libraries. To ensure homogeneity between the groups, a rebalancing procedure was employed using an iterative removal process, which was validated through analysis of variance (ANOVA) tests on all continuous variables. Differences in key physiological variables between the control and dysautonomia groups were assessed using one-way ANOVA, with Tukey’s HSD *post-hoc* tests applied for pairwise comparisons to identify significant intergroup differences. To control for the family-wise error rate, multiple comparisons were adjusted using the Bonferroni correction. Categorical variables, such as sex distribution and ergometer use, were analyzed using chi-squared tests.

### Ethical considerations

This study was reviewed by an independent institutional review board (Ethical and Independent Review Services) and was determined to be exempt due to its retrospective nature. Informed consent was obtained as a condition of clinic enrollment. The corresponding author had full access to all study data and assumes responsibility for the integrity of the data and the accuracy of the analysis.

## Results

From clinical records, 323 exercise stress tests belonging to individuals diagnosed with SLS (47), those with general OIDS (215), and healthy controls (CTL, 60) were selected for analysis and subjected to a balancing program to match age, weight, BMI, and sex. From this pool, 44 individuals with SLS, 201 with OIDS, and 53 controls were matched and subsequently selected blindly for comparison. Consistent with other published studies involving OIDS (22, 33), the vast majority of our population was female and Caucasian, even though these clinics serve regions with significant racial diversity (22, 34). Patient characteristics are summarized in Table 1.

**Table 1:** Participant demographics.

Demographic and exercise characteristics				
Parameter	Control	OIDS	SLS	*p*-value
*N*	53	210	44	
Age	18.8 ± 2.5	17.8 ± 3	18.7 ± 3.1	0.06
Weight (kg)	65 ± 14	62 ± 13	60 ± 14	0.25
BMI (kg/m^2^)	24.5 ± 4.8	23.1 ± 4.9	22.7 ± 5.8	0.13
% Female	96.2	93.8	90.9	0.55
% Ergometer use	98.1	96.7	93.3	0.40
Baseline HR	87 ± 19	95 ± 23^**^	99 ± 25^**^	0.01

### Metabolic parameters

**Figure 1:**
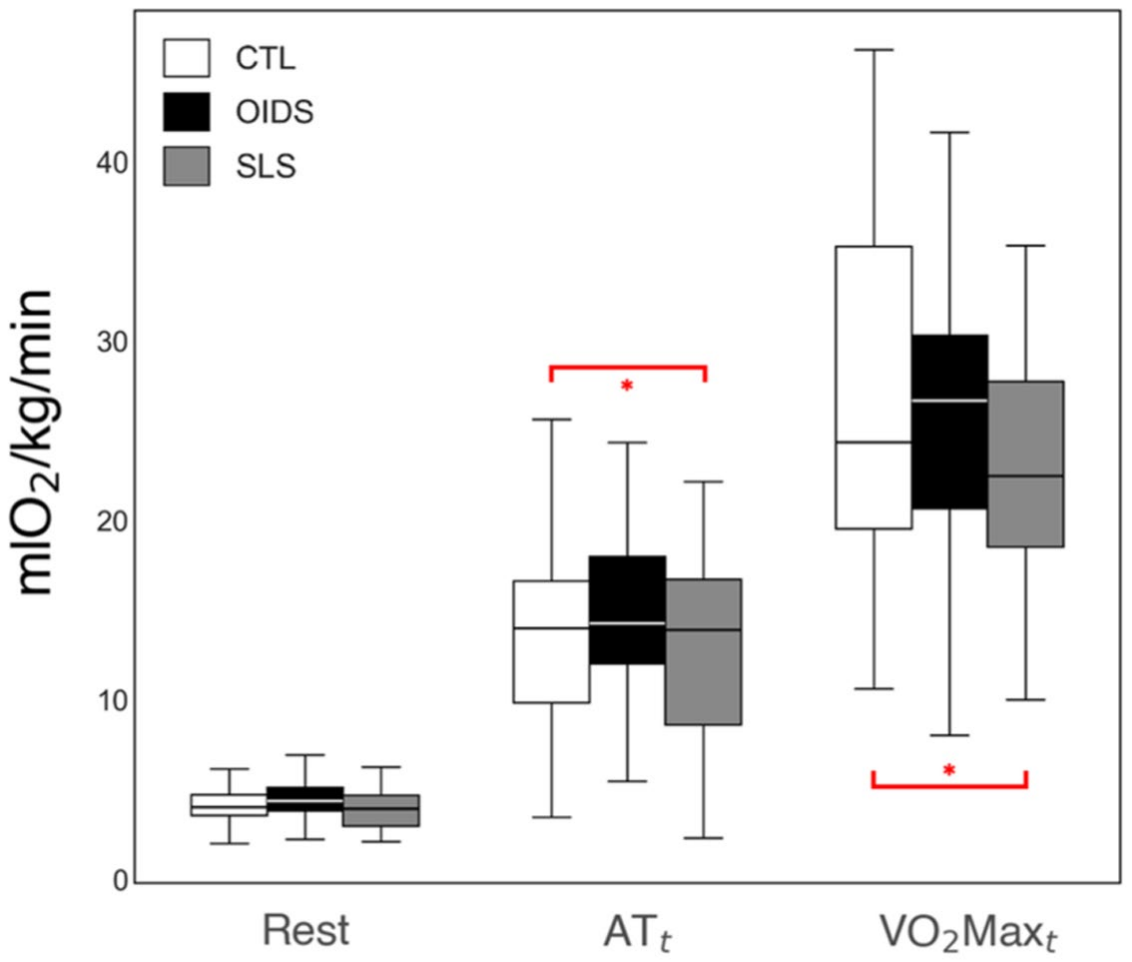
VO_2_ measures of the controls (CTL), patients with orthostatic intolerance dysautonomia syndromes (OIDS), and patients with Spiky-Leaky Syndrome (SLS) at three stages of the exercise—rest and at the times of anaerobic threshold (AT) and VO_2_max (VO_2_max_t_). Significance levels are indicated as follows: ^*^p ≤ 0.05.

Aerobic fitness is traditionally assessed using maximum oxygen consumption (VO_2_max) and anaerobic threshold (AT) as gold-standard measures. In this study, there were no significant differences in VO_2_ at rest between the three groups. However, AT and VO_2_max were significantly lower in the SLS group compared to controls, indicating impaired oxygen uptake and delivery in SLS patients (Figure 1).

**Figure 2:**
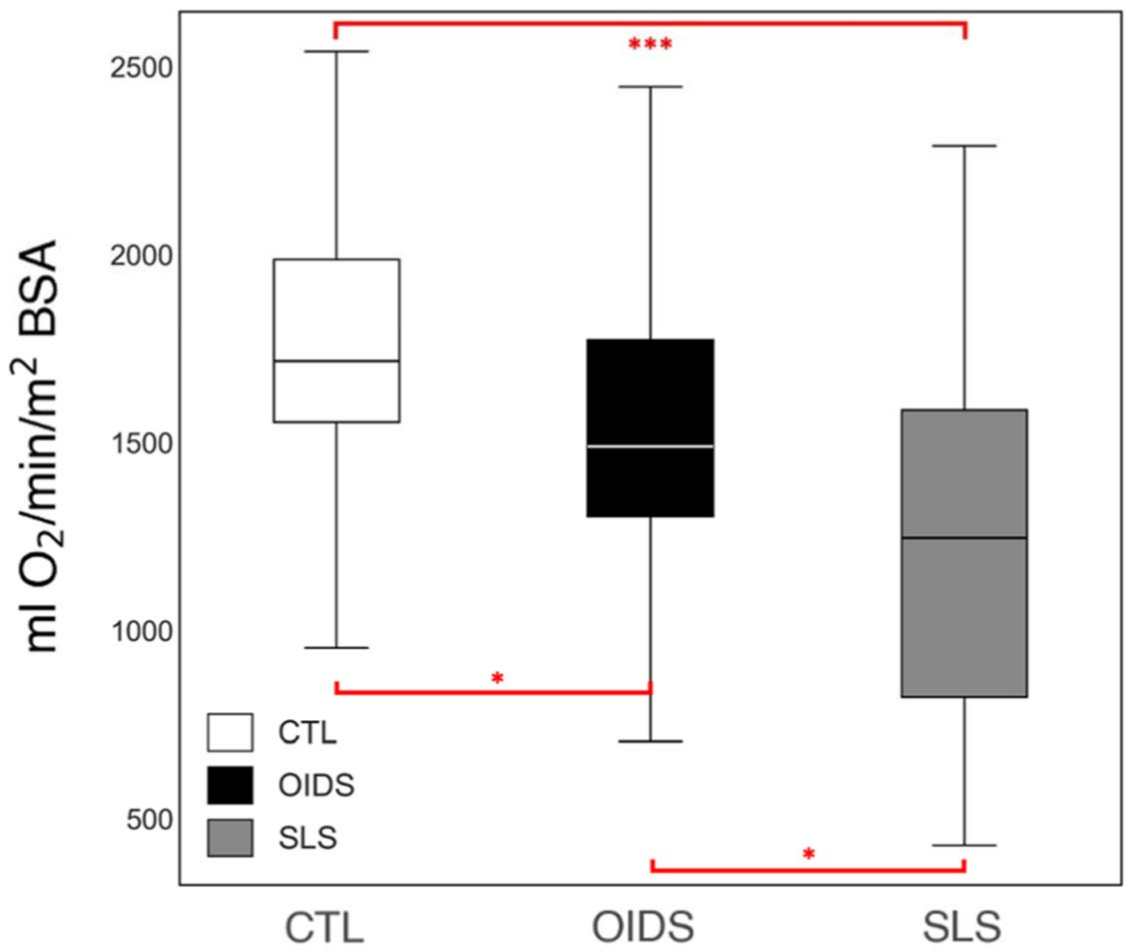
Peak oxygen uptake efficiency slope (OUESp) of the controls (CTL), patients with orthostatic dysautonomia intolerance syndromes (OIDS), and patients with Spiky-Leaky Syndrome (SLS). Significance levels are indicated as follows: ^*^p ≤ 0.05, ^***^p ≤ 0.001.

The OUESp was significantly reduced in the OIDS group compared to controls and was further reduced in the SLS group compared to the other two groups (Figure 2). This suggested a progressive decline in cardiopulmonary efficiency among patients with OIDS and SLS.

**Figure 3:**
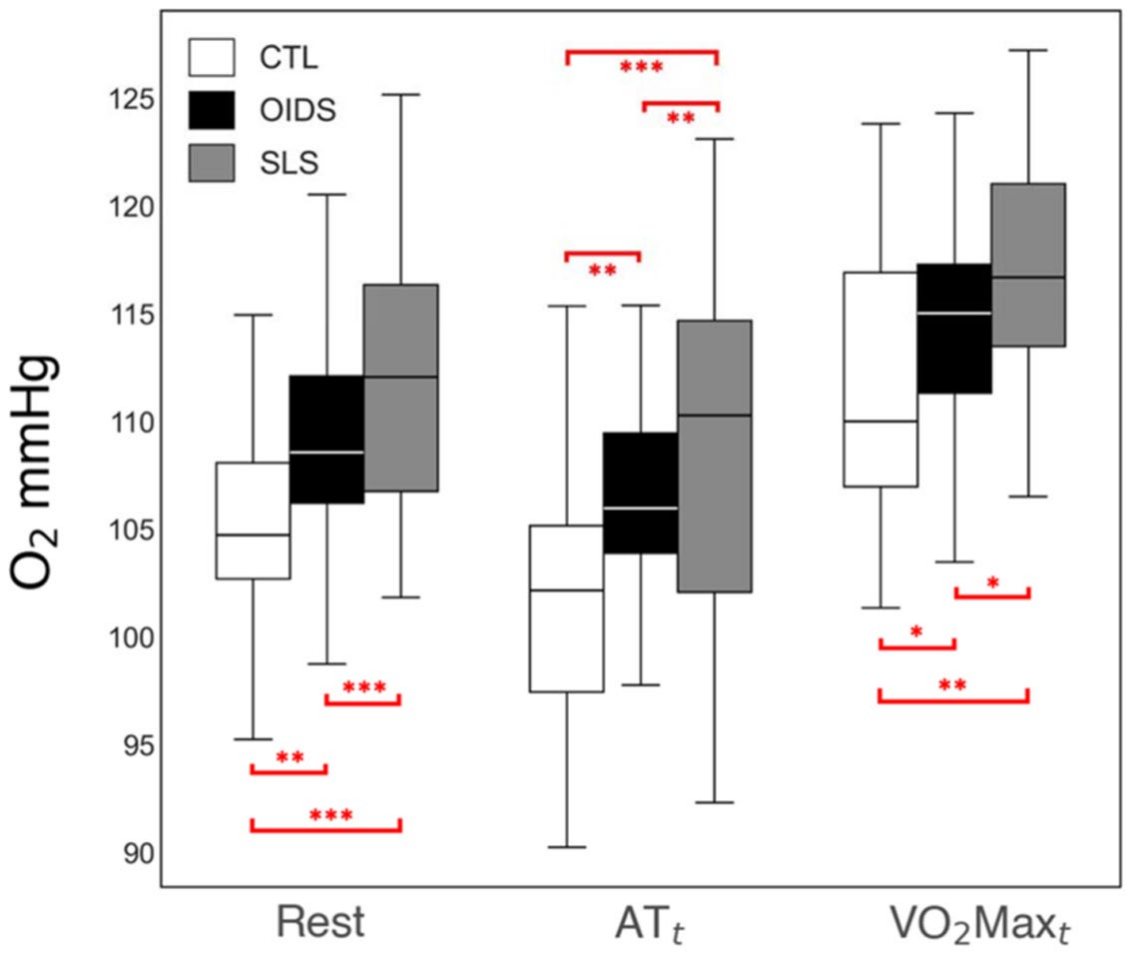
End-tidal O_2_ (ETO_2_) measures of the controls (CTL), patients with orthostatic intolerance dysautonomia syndromes (OIDS), and patients with Spiky-Leaky Syndrome (SLS) at three stages of the exercise—rest and at the times of anaerobic threshold and VO_2_max. Significance levels are indicated as follows: ^*^p ≤ 0.05, ^**^p ≤ 0.01, ^***^p ≤ 0.001.

**Figure 4:**
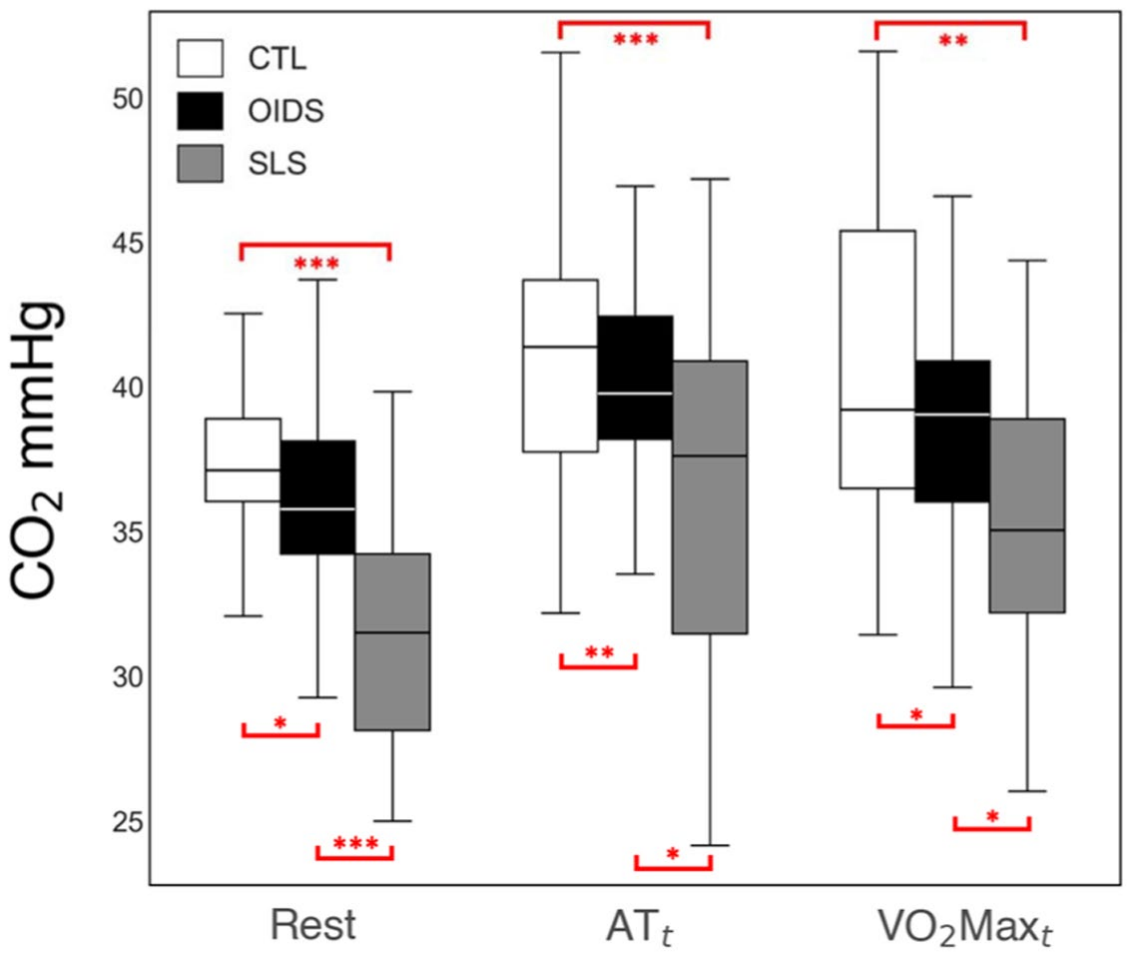
End-tidal CO_2_ (ETCO_2_) measures of the controls (CTL), patients with orthostatic intolerance dysautonomia syndromes (OIDS), and patients with Spiky-Leaky Syndrome (SLS) at three stages of the exercise—rest and at the times of anaerobic threshold and VO_2_max. Significance levels are indicated as follows: ^*^p ≤ 0.05, ^**^p ≤ 0.01, ^***^p ≤ 0.001.

ETO_2_ was elevated at all stages of exercise in OIDS patients compared to controls, with even greater elevations observed in SLS patients. The increase in ETO_2_ in SLS patients was significantly higher compared to those with OIDS, indicating a greater inefficiency in oxygen exchange. Conversely, ETCO_2_ showed an opposite trend across the three groups. ETCO_2_ levels at rest were significantly lower in SLS patients (33 ± 4 mmHg) compared to controls (38 ± 4 mmHg) and OIDS patients (36 ± 4 mmHg). This pattern was maintained across anaerobic threshold (AT_t_) and peak oxygen consumption (VO_2_max_t_), demonstrating a consistent difference in the ventilatory response (Figures 3, 4).

**Figure 5:**
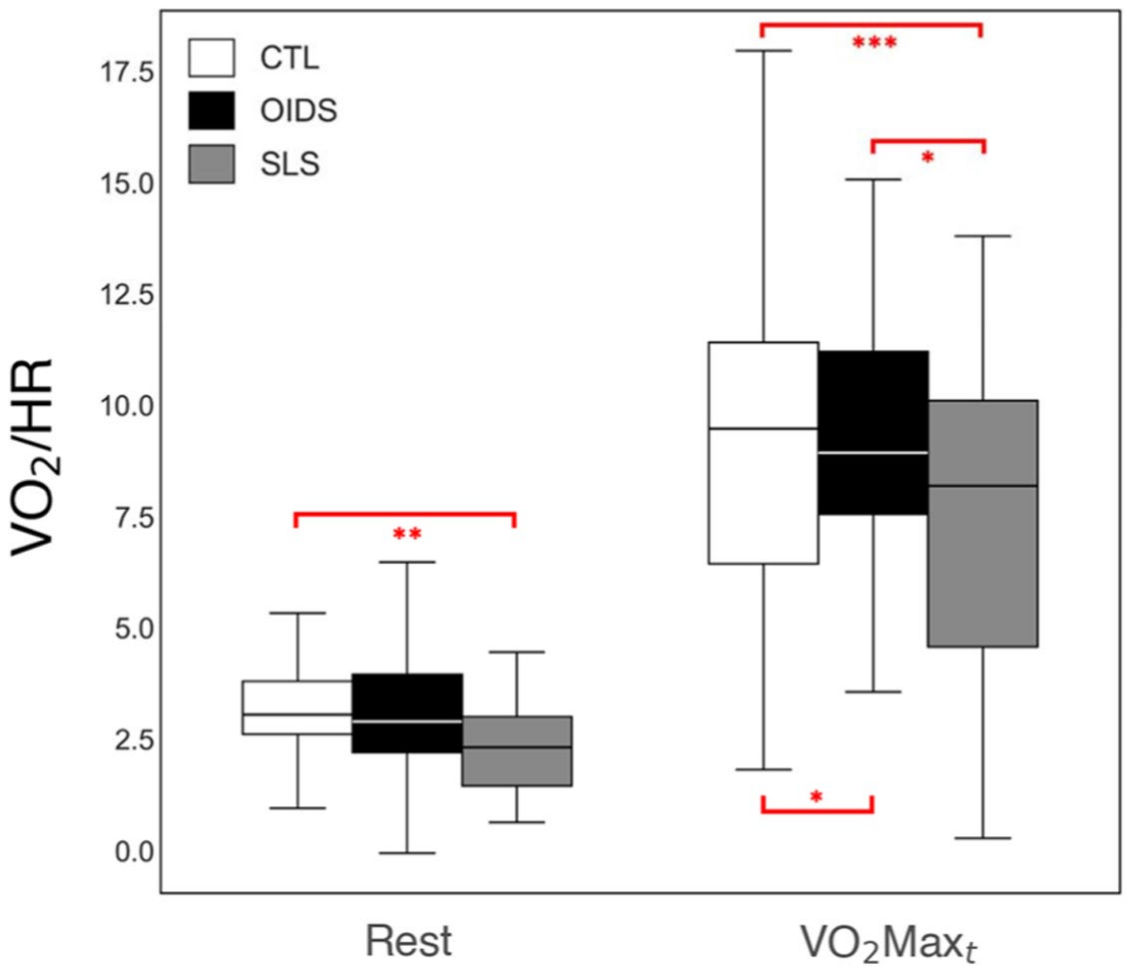
Peak oxygen pulse measures of the controls (CTL), patients with orthostatic intolerance dysautonomia syndromes (OIDS), and patients with Spiky-Leaky Syndrome (SLS) at three stages of the exercise—rest and at the times of anaerobic threshold and VO_2_max. Significance levels are indicated as follows: ^*^p ≤ 0.05, ^**^p ≤ 0.01, ^***^p ≤ 0.001. The lower oxygen pulse values during rest are a consequence of the higher baseline heart rates in the OIDS and SLS groups.

**Figure 6:**
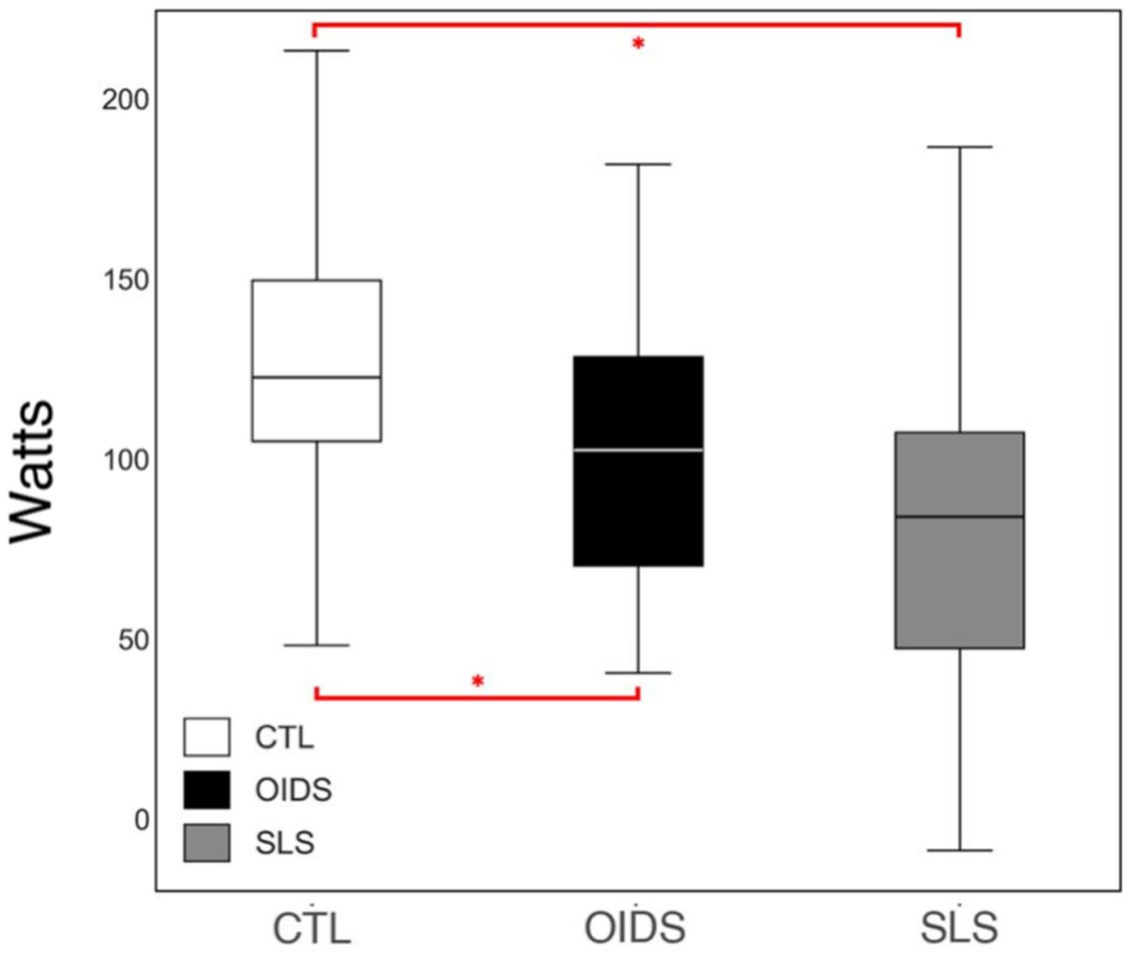
Total work performed of the controls (CTL), patients with orthostatic intolerance dysautonomia syndromes (OIDS), and patients with Spiky-Leaky Syndrome (SLS). Significance levels are indicated as follows: ^*^p ≤ 0.05.

Peak oxygen pulse, an indirect measure of stroke volume, was significantly lower in both the OIDS and SLS groups compared to the controls, with the SLS group showing the most pronounced reduction (Figure 5). In addition, when analyzing the bicycle ergometry data, total work performed was significantly lower in both OIDS and SLS groups compared to the controls (Figure 6). This reduction in exercise capacity further supports the hypothesis of impaired oxygen utilization and cardiovascular efficiency in these patient groups.

## Discussion

This study demonstrated significant reductions in VO_2_max, AT, peak oxygen pulse, total work performed, and peak oxygen uptake efficiency slope (OUESp) in patients with OIDS, with an even greater reduction observed in those with SLS. ETCO_2_ levels were significantly lower in the OIDS group at rest, at the anaerobic threshold (AT_t_), and at maximal oxygen uptake (VO_2_max_t_), with an even more pronounced decrease in the SLS group. This reduction in ETCO_2_ levels persisted throughout the exercise, following an inverse trend to ETO_2_, which was elevated in both patient groups. Among these findings, three key parameters—ETCO_2_, peak oxygen pulse, and OUESp—are particularly relevant to understanding the pathophysiology of OIDS and SLS and are discussed in detail below.

### End-tidal carbon dioxide

Among the metabolic parameters evaluated, ETCO_2_ depression throughout the entire course of the exercise is one of the most notable findings due to its potential role in assessing chronic hyperventilation and serving as a surrogate marker for sympathetic hyperactivity. A slight reduction in ETCO_2_ levels at rest is not unexpected before an exercise test, as anticipatory anxiety can induce mild hyperventilation. This likely explains why the controls had an ETCO_2_ level of 38 ± 3 mmHg, slightly lower than the expected 40 mmHg. However, the persistence of significantly reduced ETCO_2_ throughout exercise in OIDS and SLS patients suggests a sustained hyperventilatory drive. While ETCO_2_ in controls increased to 43 ± 4 mmHg during peak exercise, it remained at 37 ± 6 mmHg in those with SLS, indicating a continuous state of hyperventilation.

This abnormal ventilatory response is likely driven by two interrelated mechanisms. First, the carotid body, which plays a key role in chemoreception and ventilatory regulation, may be hyperactive as part of the sympathetic overdrive observed in these patients (5). Second, chronic respiratory alkalosis, induced by persistent daytime hyperventilation, is likely compensated by renal metabolic acidosis, which maintains blood pH homeostasis. Evidence for this renal correction was not extracted from the records and remains a subject for future studies. In the context of SLS, this chronic hypocarbia may have profound effects on cerebral blood flow, leading to a reduction in cerebral perfusion during waking hours. At night, airway obstruction and resultant hypercarbia could induce a rapid increase in cerebral blood flow, leading to spikes in CSF pressure (14).

### Peak oxygen pulse

Peak oxygen pulse, which serves as a surrogate for stroke volume, was another critical metabolic parameter in this study. It is widely used in assessing cardiovascular function, particularly in pediatric and congenital heart disease populations, as well as in patients with autonomic dysfunction. Athletic training enhances oxygen pulse, while deconditioning flattens the oxygen pulse curve (35). A reduction in oxygen pulse is associated with diminished stroke volume, which can result from impaired myocardial contractility, reduced left ventricular (LV) chamber size, or decreased ventricular filling volume (36).

**Figure 7:**
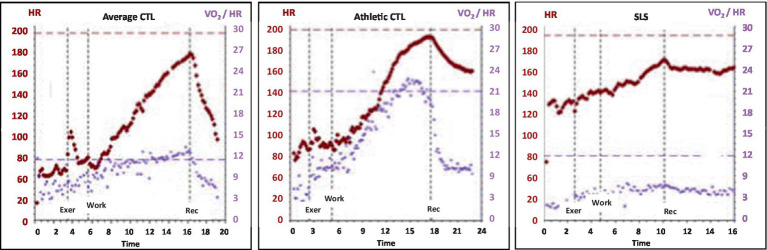
Oxygen pulse curves of the patients through the course of the exercise, showing those of a control with average fitness, (Average CTL), a control with athletic fitness (Athletic CTL), and a patient with Spiky-Leaky Syndrome (SLS). The curve rises to peak exercise, with a peak oxygen pulse of approximately 11 mlO2/beat in a healthy person with average fitness. It rises to 22 in a person who is aerobically trained, and it is depressed at all levels of exercise in a person with POTS physiology.

In the context of OIDS and SLS, a flattened oxygen pulse curve was expected, as it mirrors the low-flow state commonly observed in POTS, where reduced ventricular filling leads to low stroke volume (37). Given this, the significantly reduced oxygen pulse in OIDS patients, regardless of whether they met POTS criteria, was not surprising. Had the OIDS cohort been restricted to patients formally diagnosed with POTS, and if POTS was not fully managed at the time of testing, the reduction would likely have been even more pronounced. The SLS group exhibited an even flatter oxygen pulse curve than the OIDS group, further supporting the presence of compromised stroke volume and impaired cardiovascular efficiency in these patients. This may simply be a consequence of the higher prevalence of POTS patients in our SLS cohort compared to the general OIDS cohort (~ 65% vs. 25%, respectively). Figure 7 illustrates the curve of a typical healthy person and two extremes from this study.

### Peak oxygen uptake efficiency slope

**Figure 8:**
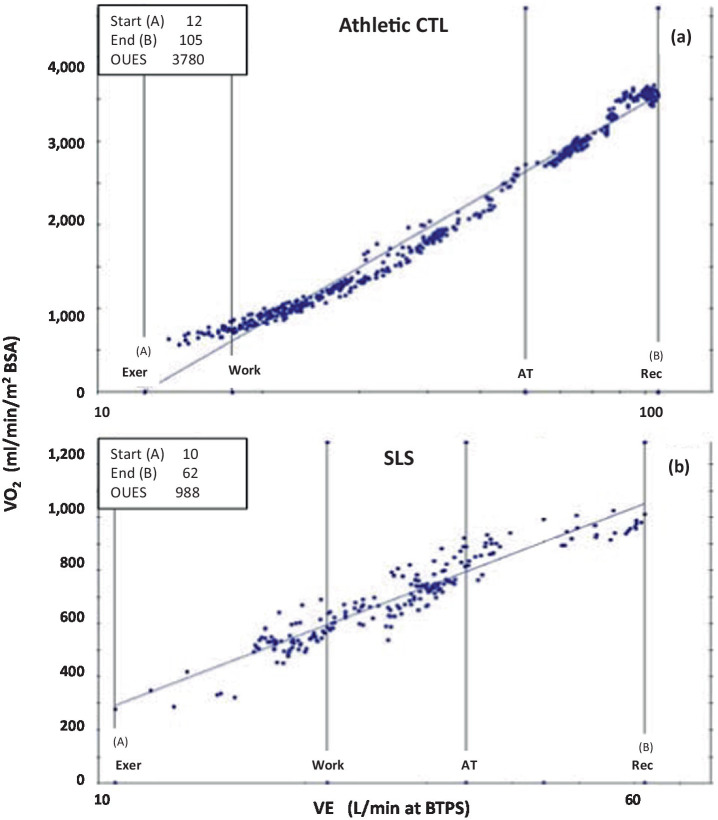
Oxygen Uptake Efficiency Slopes of two extremes from this study; **(a)** that of a control patient with athletic fitness (Athletic CTL), and **(b)** of a patient with Spiky-Leaky Syndrome (SLS). x axis: Minute Ventilation VE at BTPS (body temperature (37C, ambient pressure and gas saturated with water vapor), y axis: Oxygen consumption normalized to BSA. Note that the scales of the two Y axes are quite different resulting are much different slopes. Normal values for OUESp is 1,400 to 2,000 ml O2/min/m^2^BSA. An aerobically-trained healthy athlete can approach 4,000 while those with cardiovascular compromise may fall below 1,400 ml O2/min/m^2^BSA. Normal values for OUESp is 1,000 to 2,200 mL O2/min/m^2^BSA (31, 32).

Recent advancements in exercise physiology have introduced improved methods for assessing peak fitness, particularly in individuals with congenital heart disease (32). One such metric, the oxygen uptake efficiency slope (OUES), was first introduced by Baba et al. (38) and later refined for clinical application by Meucci (31). The OUES is considered an effort-independent measure of exercise capacity, making it particularly useful for populations in which maximal exertion may not be achievable. The OUES is calculated as the best-fit slope of the logarithmic (base 10) relationship between minute ventilation (VE) and oxygen uptake (VO_2_). A steeper slope indicates greater exercise capacity. Meucci recently demonstrated that the peak oxygen uptake efficiency slope (OUESp) correlates strongly with peak VO_2_ and follows a linear increase with age throughout childhood and adolescence (31, 39, 40). This makes the OUESp particularly valuable for assessing patients who terminate exercise early, before reaching their peak heart rate, such as those with OIDS. Given the underlying physiological impairments in OIDS and SLS, a reduction in the OUESp is expected. Indeed, most SLS cases in this study exhibited OUESp values ranging from 1,000 to 1,600 mlO_2_/min/m^2^ BSA, placing them between normal averages and the lower limit of normal, as defined by Meucci (31). Figure 8 illustrates two extreme examples from this study, further demonstrating the reduced OUESp in the SLS population.

### Limitations

This study was a retrospective review and carried the inherent limitations of such a design. To minimize selection bias, researchers were blinded to group categorization and data outcomes when selecting cases for the original database. In addition, the final dataset was subjected to a balancing program that was also blinded to the outcomes, further reducing the potential bias in selection.

The study population was drawn from five clinics within a limited geographic area, all under the care of a single complex care cardiovascular specialist. As a result, geographic, environmental, and referral biases likely influenced the sample, favoring patients with OIDS, HSD, and MCAS over typical POTS and straightforward OIDS cases. One notable effect of this referral bias is the absence of neurogenic orthostatic hypotension (nOH) as a primary presentation, as such patients are less likely to be referred to a cardiologist or remain under the care of their primary care physician. In addition, the decision to include a broader dysautonomia population rather than focusing solely on POTS might have impacted the results. This approach aligns with that of Wheeler et al. (1), who emphasized the need to assess dysautonomia patients who do not meet POTS criteria. Our broader selection better reflects the SLS population, which, like OIDS, often presents without POTS. However, 25% of OIDS patients and 65% of SLS patients in this study met the formal POTS criteria.

Another limitation is the reliance on surrogate measures, a necessary consequence of using non-invasive data collected during routine patient care. In this study, ETCO_2_ served as a surrogate for hypocapnia during the exercise, which, in turn, was used as a proxy for hypocarbia and postulated as a potential objective marker of sympathetic hyperactivity. Similarly, oxygen pulse was used as a surrogate for stroke volume, which itself was considered a proxy for preload and, therefore, a marker of POTS physiology.

## Conclusion

Patients with OIDS exhibit reduced metabolic exercise parameters and frequently display flattened oxygen pulse curves, suggesting reduced ventricular stroke volume. This reduction may be due to insufficient cardiac preload, a hallmark of POTS physiology, regardless of whether tachycardia is present. Although the underlying mechanisms remain unclear, many of these patients also develop persistently low levels of ETCO_2_, both at rest and during exercise, indicating a pattern of chronic hyperventilation. These two exercise parameters—peak oxygen pulse and ETCO_2_—may serve as objective markers for POTS physiology and sympathetic hyperactivity, respectively. Patients diagnosed with Spiky-Leaky Syndrome (SLS) exhibit even more pronounced reductions in ventricular stroke volume and awake hyperventilation, as measured by these same parameters. We postulate that these physiological abnormalities contribute to episodic spikes in cerebrospinal fluid (CSF) pressure, leading to intermittent occult CSF leaks, as outlined in our recent description of SLS (14).

## Data Availability

The raw data supporting the conclusions of this article will be made available by the authors, without undue reservation.
